# Cardiovascular Disease Risk Factors: Hypertension, Diabetes Mellitus and Obesity among Tabuk Citizens in Saudi Arabia

**DOI:** 10.2174/1874192401812010041

**Published:** 2018-04-23

**Authors:** Jeneth Gutierrez, Aladeen Alloubani, Mohammad Mari, Mohammad Alzaatreh

**Affiliations:** 1 Nursing Department, University of Tabuk, Tabuk, Saudi Arabia; 2 Nursing Research Unit, King Hussein Cancer Center, Amman, Jordan; 3 School of Nursing, University of Jordan, Amman, Jordan

**Keywords:** Cardiovascular disease, Risk factors, Hypertension, Diabetes mellitus, Obesity, Body mass index

## Abstract

**Background:**

Cardiovascular Disease (CVD) is considered as the main cause of death worldwide. Identifying the links among CVDs risk factors can help decrease CVD-related deaths.

**Aim:**

To assess the prevalence of risk factors for CVD and their relationships among the Tabuk City population in Saudi Arabia.

**Methods:**

A cross-sectional design was used; 432 participants in the Tabuk region were included in this study.

**Results:**

The prevalence of diabetes mellitus (DM) was 5.6%, the prevalence of hypertension (HTN) was 11.1% and obesity and overweight together were 69.9%. Mean Body Mass Index (BMI), HTN, and DM increased with age. There was a correlation between BMI with HTN (r=.200, p<.001), BMI and DM (r=.149, p<.001) and DM and HTN (r=.366, p<.001).

**Conclusion:**

Public awareness may help in reducing the prevalence of CVD.

## 1 INTRODUCTION

Cardiovascular Disease (CVD) is the major worldwide Non-Communicable Disease (NCD). There will be growing burden of CVD in developing countries after few decades [1]. This is related to the social and socioeconomic changes in developing countries. Many NCDs can be limited by preventing risk factors. Globally, the main cause of death is CVD [2-4]. According to the Canadian Heart and Stroke Foundation age, sex, family history, tobacco smoking, physical inactivity, unhealthy diet and obesity are some significant risk factors that cause CVD [5]. Minimising the prevalence of behavioural CVD risk factors can prevent CVD [6].

During 2015, NCDs caused about 39.5 million deaths out of 56.4 million of total deaths in the world; CVD alone was responsible for 17.7 million deaths [7]. Additionally, in low and middle-income countries, approximately 30.7 million, *i.e.* three-quarter of deaths occurred due to NCDs [7]. CVD is responsible for global growing rates of morbidity and mortality in adults according to the World Health Organisation (WHO) [8]. Improving general physical, social, emotional and spiritual welfare should be included to upgrade a person’s health because it has a huge effect on family life. Moreover, in Saudi Arabia, CVD contributes an immense load on the health care organizations and workers [9].

In Saudi Arabia, research was carried out at King Faisal University to assess the prevalence of coronary heart disease risk factors among 159 male students [10]. Practising of physical activity was not observed by 28.9% students, on the other hand, watching TV and using a computer for hours were observed in 37.7% and 46.5% students, respectively, smoking in 19%, overweight in 24.5%, obesity in 11.9% and severe obesity in 10.7% based on Body Mass Index (BMI) and waist-hip ratio. Mean systolic and diastolic blood pressure readings of 122.8 and 71.5 mmHg, respectively, were observed in students [10]. A review study related to physical inactivity in the Saudi Arabian population discusses its implications for health, the data showed 43.3-99.5% total inactivity rate among adults and children [11].

In Bahrain, complex CVD risk factors were observed in a cross-sectional study showing 78.4% total prevalence rate of overweight and obesity; for participants who did not perform any physical activity it was 50.8%. In addition, on a daily basis, fruits and vegetables were not eaten by 24.3%, and recent smoking was observed in 16.1%. No less than 3 CVD risk factors were observed in the majority of participants (95.35%) and 3-5 risk factors were observed in 4.65% of the participants [12]. In 2007, a 36.3% prevalence of obesity and 32.9% prevalence of overweight was reported by the Bahraini National NCD Risk Factors Survey [13]. Moreover, daily intake of vegetables was recorded 62% while intake of fruits was recorded in only 49.6%. No involvement in physical activity in free time was noted in about 57.1%. Lastly, 19.9% were smokers [13].

In the United Arab Emirates, cross-sectional community-based research was conducted to identify established CVD risk factors by means of the Framingham risk score for risk assessment [14]. According to this research, 28.4% of the participants had a score >20%, Diabetes Mellitus (DM) was observed in 23.3%, hypertension (HTN) in 20.8%, obesity in 37.3%, metabolic syndrome in 22.7% and smoking habit was observed in 19.6% of the male participants; 2.4% of the participants were found to have coronary heart disease, and 64% males and 53% females had an abnormal lipid profile mainly because of Low-Density Lipoprotein Cholesterol (LDL-C) or triglycerides levels [14].

In Iran, cross-sectional research [15] was carried out regarding CVD risk factors; 36.6% males and 35.9% females were overweight. Obesity was observed in 11.2% males and 28.1% females. Increase in the mean BMI, waist circumference, and the waist-hip ratio was observed up to the age of 65 years. As risk factors, males and females had increased serum cholesterol, triglycerides, glucose level (2 h after drinking a glucose solution), waist circumference and BMI [15].

It is clear from a long-term follow-up research that after 65 years of age a prolonged, healthier life and decreased medical care expenses are related to a favourable risk factor profile during the working years [16]. A considerable decline in the major adverse cardiovascular events was also observed due to the treatment of each of the major cardiovascular risk factors [16].

## 2 AIMS

To assess the prevalence of risk factors for CVD among the Tabuk City population in Saudi Arabia. We also assessed the correlation among the risk factors of coronary artery disease including BMI, HTN and DM to establish baseline data for upcoming programs.

## 3 METHODOLOGY

### 3.1 Setting and Sample

A non-experimental, cross-sectional design was used. This research was conducted in Tabuk City in Saudi Arabia. Tabuk is one of the 13 major regions in Saudi Arabia located in the north western area of the Kingdom with a total population of around 571,000 [17].

Data were collected at the big major malls to ensure the representation of the population. A sample of 432 participants was collected. The sample size was estimated by using the Slovin’s formula based on a power of .80, level of significant .05, population size (n) 571,000 and a medium effect size. The required sample size based on that was at least 400 participants. In the study, 432 participants were included to overcome any attrition during data collection process [18]. Inclusion criteria were all people who live in Tabuk City and with an age ≥18 years old.

### 3.2 Methods

A self-administered structured questionnaire was used in this study. Validity and reliability were tested in a previous study based on a sample size of 120. The questionnaire was made from multiple-choice questions that needed about 10 min to finish. The questionnaire included demographic variables like age, gender, nationality, marital status, educational level, occupation and income. Anthropometric measures including blood pressure, Random Blood Sugar (RBS), height, weight and BMI, were also recorded.

Data were collected by the authors and trained nursing and medical students in their internship in October 2016. The participants were voluntarily involved in this study after explaining the objectives, significance, confidential use of data, and utilization of the outcomes. The respondents were advised about their right to either agree to participate, withdraw, or decline without any risk or benefits.

Anthropometric measurements were obtained by trained nursing and medical students in their internship. The participants were asked to take 10 min rest, and then, blood pressure was measured 2 times in sitting position at an interval of 10 min using mercury sphygmomanometers. The mean of these 2 readings was considered for analysis. Blood pressure readings were categorized according to American Heart Association (AHA) into 4 groups: in which 1-4 scores were assigned, score 1 means that the participant has normal blood pressure range (systolic reading <120 and/or diastolic <80), a score of 2 represents prehypertension (systolic reading and/or 120-139, diastolic 80-89), while a score 3 demonstrates high blood pressure stage 1 (systolic reading 140-159 and/or diastolic 90-99), and finally score 4 represents stage 2 (systolic reading >160 and/or diastolic <100) [19]. Weight scales with stadiometers were used to measure weight and height, and BMI was calculated based on the formula BMI were classified into 4 categories: <18.5 means underweight, 18.5 to <25 means normal weight, 25 to <30 is overweight and >30 is considered obese [20]. RBS was measured by a glucometer (Accu-Chek® Active Blood Glucose Meter) and the reading was subdivided into 2 groups; normal (<200 mg/dl); and hyperglycaemia (≥200 mg/dl) [21]. During their interview, the participants were provided with their blood pressure, RBS, height, weight, and BMI results.

### 3.3 Ethical Consideration

This study was intended to protect human rights. The details of the study were fully disclosed in the informed consent. The researchers were available at the time of consent and throughout the study to answer questions from the participants. Strategies were utilized to protect the participant data. At any time during the study, the participants were allowed to voluntarily quit the study. The ethical approval was obtained from the University of Tabuk Ethical Committee before the beginning of data collection. All information collected during this study was kept confidential. Each participant was assigned a study serial number to ensure the confidentiality and privacy.

### 3.4 Data Analysis

The Statistical Package for the Social Sciences (SPSS) (version 23) was used to analyse the results. Descriptive statistics were used to analyse demographic data, whereas inferential statistics (*t*-test and Spearman’s rho) were utilized to assess the relationships between demographics and the dependent variables. The Pearson product-moment correlation coefficient (Pearson’s correlation, for short) was used to measure the strength and direction of relationship between BMI, HTN and DM.

## 4 RESULTS

### 4.1 Demographic Characteristics

The total number of participants was 432; males 235 (54.4%) and females 197 (45.6%). Most of the participants were 18-30 years old. This group involved 208 participants (48.1%) of the sample. The second group was those with age ranging from 31-50 years including 129 participants (29.9%) of the total sample. The last group was those above 50 years old. This group had 95 participants (22% of the total sample).

The education level was categorized into 4 levels: primary, secondary, bachelor degree and higher education. The bachelor's group was predominant with a total number of 170 individuals (39.3%) in the overall sample. The secondary education level group had a total number of 162 participants (37.5%). Information relating to marital status was categorized into single, married, divorced or widowed. The single group had 158 participants (36.6%), the married group had 267 (61.8%) participants, and the divorced and widow groups were only 3 (0.7%) and 4 (0.9%), respectively.

Other demographic elements considered were nationality and income status; 386 (89.4%) of the participants were Saudi and 46 (10.6%) were Non-Saudi. The income section revealed that the participants whose earning was <5000 Saudi Riyal (SR) were 167 (38.7%). Also, those who had an income between 5000-10000 SR were 163 (37.7%). Finally, those individuals whose income was >10000 SR were 102 (23.6%). Detailed demographic data of the study sample is displayed in Table **1**.

### 4.2 Prevalence of Obesity, HTN, DM

If the participant had a BMI between 25 and 30 Kg/m^2^, he/she was considered overweight, and if the BMI was >30 he/she was considered obese. Total number of overweight and obese participants was 302 (69.9%), which indicates a high percentage of obesity in the sample, Nonetheless, the mean BMI was 28.3 Kg/m^2^. Hypertensive participants were 48 (11.1%), however, pre-hypertensive participants (systolic pressure 120-139 and/or diastolic 80-89 mmHg) were 80 (18.5%). RBS was used as a DM criterion, patients with hyperglycaemia (RBS ≥ 200 mg/dl) were 24 (5.6%). RBS ranged from 72 to 417 mg/dl; the mean was 117 mg/dl (standard deviation 48).

### 4.3 BMI, HTN and DM Differences by Gender

Independent sample t-test was used to assess the differences between males and females in terms of BMI and the presence of DM. RBS level was significantly different between males and females; males 122 mg/dl and females 111 mg/dl (*p =*.015). Other details are displayed in Table **2**. Furthermore, data related to HTN were analysed by Chi-square. More men had a higher blood pressure than women (p <.001). Other details are presented in Table **3**.

### 4.4 BMI and DM Differences Per Age

**Fig. (1):**
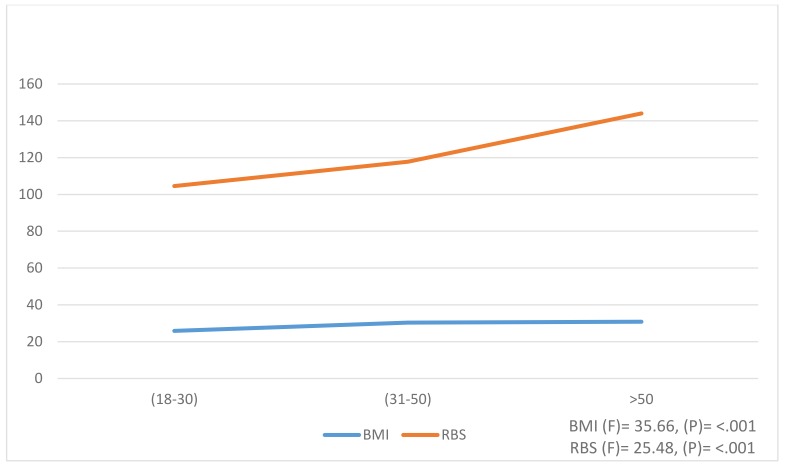
ANOVA per age with Body Mass Index & Random Blood Sugar.

ANOVA analysis was conducted to evaluate the differences in BMI and RBS variables relating to age groups (Fig. **1**). Overall, there were significant differences among age groups in the 2 variables. There was a slight increase in the mean of BMI as age progressed. For example, participants of age above 50 years had the highest level of BMI (mean 30.8 ± 5.0) while the groups of 31-50 years and 18-30 years had BMI means 30.3 ± 6.5 and 25.9 ± 5.6, respectively at p <.001. In addition, the RBS increased significantly at p-value < .001 for all the 3 age groups.

### 4.5 HTN Differences Per Age

The Spearman rank-order correlation coefficient analysis was conducted to assess the strength and direction of the relationship between HTN and age groups. There was a positive significant relationship between age and HTN (rs= .289**, p= < .001). Generally, as age advanced there was an increase in blood pressure. Detailed data are presented in Table **4**.

### 4.6 Correlation Among the BMI, HTN and DM

This section shows a summary of the relation among BMI, HTN and DM. Table **5**. displays the correlation among these 3 variables by a Pearson product-moment correlation. A positive correlation was detected among BMI with HTN and DM (*r* = .200** and .149**, respectively at *p*<.001), whereas the relationship between HTN and DM was (*r* = .366**, *p*<.001). Indeed, this relationship was the strongest correlation observed among these 3 variables.

## 5 DISCUSSION

According to Kontis **et al.** [22], there has been a high prevalence of cases of cardiovascular health problems worldwide. These cases are attributed to risk factors such as HTN, obesity and overweight, BMI of >25, physical inactivity and lack of adequate fruit consumption [23, 24]. Lifestyle and some administrative jobs have been shown to be potential risk factors for obesity and overweight especially [25].

The results obtained show an alarming rate of some risk factors. According to this study, there is an increase in the cases of DM, HTN and BMI as age increases. This is probably because as individuals age, they tend to participate less in physical activities which are essential to reduce sugar levels [26]. Moreover, the economic transition in Saudi Arabia has prompted potential influences on individual’s lifestyle. For example, it has contributed to occupational patterns that are detrimental to most of the physical activities [27].

This study reported that HTN affects 11.1% of the participants, which is consistent with a previous study in Saudi Arabia [28]. This reveals that the global HTN burden is widespread and growing, signifying the necessity for intervention to prevent this disease. Furthermore, the findings recognized a significant association between HTN and age in males and females, which is in line with national and worldwide studies on various populations with diverse geographical, environmental, social and financial attributes [29, 30].

Internationally, the prevalence rate of DM is expected to increase by up to 42% from 2003 to 2025 [31]. The prevalence in the Gulf area is highest in Bahrain (25.7%) and Oman (16.1%) [32, 33]. This study showed an additional growth in the prevalence of DM in previous studies conducted in Saudi Arabia. This study displayed an increase in the blood sugar level as age increases. Studies carried out in Saudi Arabia showed diverse age-specific prevalence rates. Certainly, the association between DM and age is consistent with previous studies [34, 35]. The recent reports of the International Diabetes Federation about the prevalence of DM showed comparable outcomes [36]. The results of this study indicate that participants of age above 50 years had considerable problems in glucose level, which coincides with World Health Organization’s report; 51% of aged people suffer from abnormal glucose level [2, 37].

Globally, the prevalence of DM is comparable in both genders [38, 39], but it is slightly higher in females than in males at age <60 years [40], but in the present study, men had a higher RBS than women. Al-Nozha **et al.** [34] in 2004, documented a 20% prevalence of DM in the Saudi Arabia population in both genders. Another study [41] in 1987, stated that the rate of DM is similar in both genders in a rural area in Saudi Arabia; both studies were inconsistent with the present study. One reason may be a change in patterns over the years.

DM is reported to increase as age increases [42, 43]. The prevalence of DM was greater in males compared with females (p =.015). This clearly provides a strong rationale for the increasing prevalence of cardiovascular health problems in Saudi Arabia.

The findings of this study showed that men who took part in this study had a lower rate of obesity compared with women. This finding was inconsistent with the result of studies by Ferdinandy **et al.** [44] in 2014 and Jarrah **et al.** [45] in 2011, which showed that the mean BMI of women was significantly lower than that of men. The finding of this study may reflect a rationale that women tend to be more inactive than men.

### CONCLUSION AND RECOMMENDATIONS

Several risk factors were identified to contribute in CVD in Saudi Arabia. The findings of this study pointed out that as the magnitude of the BMI increases, the positive association with the risk factors also increases. Another important consideration is weight management, a fundamental step in countering cardiovascular health problems. Some of the weight loss activities that were found to be useful include: dietary pattern management and physical activities among others. One of the major identified recommendations is the creation of public awareness through campaigns to aid in reducing the prevalence of CVD. The study recommends that a larger study covering all the aspects of risk factors associated with CVD should be conducted. Besides, the government should encourage country-wide awareness of the risk factors through campaigns and increase the service quality provided in hospitals to boost better understanding and management of overweight, DM and HTN to prevent CVD.

## Figures and Tables

**Table 1:** Demographic characteristics of the sample.

Variable	*N*	%
**Gender**	–	
Male	235	54.4
Female	197	45.6
**Age (years)**	–	
(18-30)	208	48.1
(31-50)	129	29.9
(> 50)	95	22
**Level of education**	–	
Primary	92	21.3
Secondary	162	37.5
Bachelor degree	170	39.35
Higher education	8	1.85
**Marital Status**	–	
Single	158	36.6
Married	267	61.8
Divorced	3	.7
Widow	4	.9
**Nationality**	–	
Saudi	386	89.4
Non-Saudi	46	10.6
**Income (Saudi Riyals)**	–	
< 5000	167	38.7
5000-1000	163	37.7
> 1000	102	23.6

**Table 2:** Gender, Body Mass Index and Diabetes Mellitus.

Gender	Male *Mean (SD)*	Female *Mean (SD)*		p *(t-test)*	
BMI	27.9 (5.8)	28.7 (6.7)		.67 (1.3)	
RBS	122 (54)	111 (38)		.015 (2.4)	

BMI: Body Mass Index, RBS: Random Blood Sugar

**Table 3:** Chi-square per gender with hypertension.

Gender	Normal Blood Pressure *n=304 (%)*	Prehypertension *n=80 (%)*	Hypertension Stage 1 *n=41 (%)*	Hypertension Stage 2 *n=7 (%)*	p (χ2 test)
Male	136 (44.7)	58 (72.5)	34 (82.9)	7 (100)	<.001 (41.3)
Female	168 (55.3)	22 (27.5)	7 (17.1)	0 (0)	

**Table 4:** Spearman’s rho per age with Hypertension.

Age	Normal Blood Pressure *n=304 (%)*	Prehypertension *n=80 (%)*	Hypertension Stage 1 *n=41 (%)*	Hypertension Stage 2 *n=7 (%)*	r_s_	p
(18-30)	170 (55.9)	31 (38.75)	7 (17.1)	0 (0)	.289**	<.001
(31-50)	85 (28)	28 (35)	16 (39)	0 (0)		
>50	49 (16.1)	21 (26.25)	18 (43.9)	7 (100)		

**Table 5:** Correlation among Body Mass Index, Hypertension & Diabetes Mellitus.

–	*BMI*	*HTN*	*DM*
*BMI r*	*1*	.200**	.149**
p value	–	*<.001*	*< .001*
*HTN r*	.200**	*1*	.366**
p value	*< .001*	–	*< .001*
*DM r*	.149**	.366**	*1*
p value	*< .001*	*< .001*	–

BMI: Body Mass Index, HTN: Hypertension, DM: Diabetes Mellitus **Correlation is significant at the 0.01 level (2-tailed).
